# Clinical utility of smartphone-based audiometry for early hearing loss detection in HIV-positive children: A feasibility study

**DOI:** 10.4102/phcfm.v13i1.3077

**Published:** 2021-09-30

**Authors:** Mukovhe Phanguphangu, Andrew J. Ross

**Affiliations:** 1 Department of Family Medicine, Faculty of Health Sciences, University of KwaZulu-Natal, Durban, South Africa; 2 Department of Rehabilitative Sciences, Faculty of Health Sciences, University of Fort Hare, East London, South Africa

**Keywords:** paediatric HIV, hearing loss, early detection, self-administered, mHealth

## Abstract

**Background:**

Paediatric human immunodeficiency virus (HIV)/acquired immune deficiency syndrome (AIDS) often manifests with hearing loss (HL). Given the impact of HL, early detection is critical to prevent its associated effects. Yet, the majority of children living with HIV/AIDS (CLWHA) cannot access hearing healthcare services because of the scarcity of audiologists and expensive costs of purchasing screening equipment. Alternative solutions for early detection of HL are therefore necessary.

**Aim:**

The overall aim of this study was to assess the feasibility of using self-administered smartphone-based audiometry for early HL detection amongst CLWHA.

**Setting:**

This study was conducted at the paediatrics department of a state hospital in the Eastern Cape province, South Africa.

**Methods:**

This was a feasibility study conducted amongst twenty-seven (27) CLWHA who were in the age group of 6–12 years. The participants self-administered hearing screening tests using a smartphone-based audiometric test. The primary end-points of this study were to determine the sensitivity, specificity and test-retest reliability of self-administered hearing screening.

**Results:**

The sensitivity and specificity for self-administered screening were 82% and 94%, respectively, with positive and negative predictive values of 90% and 88%, respectively. Moreover, a strong positive test-retest reliability (*r* = 0.97) was obtained when participants self-administered the screening test.

**Conclusion:**

Six- to 12-year-old CLWHA were able to accurately self-administer hearing screening tests using smartphone-based audiometry. These findings show that self-administered smartphone audiometry can be used for serial hearing monitoring in at-risk paediatric patients.

## Introduction

An estimated 240 000 children are living with the human immunodeficiency virus (HIV)/acquired immune deficiency syndrome (AIDS) in South Africa (SA).^1^ Unfortunately, paediatric HIV/AIDS often occurs with a high prevalence of hearing loss (HL).^2,3,4^ A recent study in Mthatha^2^ reported the prevalence of HL in 32% of the participants whilst a Durban study^3^ reported a 51% HL prevalence, both in children who are in the age group of 5–12 years. In Cape Town, the prevalence of HL in children living with HIV/AIDS (CLWHA) aged below 14 years was estimated at 16% (*n* = 5/37).^4^ The high prevalence of HL in children at this age group is of concern given the impact that HL has on childhood development. For instance, HL during childhood negatively impacts communication skills, socio-emotional development and scholastic performance.^5,6^ The latter leads to poor academic achievement, which often translates to poor vocational outcomes and dependence on social security.^6^ Furthermore, HL also leads to poor socio-emotional outcomes and bullying, which result in social isolation, behavioural problems and an overall poor quality of life.^7,8,9,10^ Early detection and intervention (EDI) of HL are therefore important to prevent these negative impacts.^6^ This is particularly important because more than 60% of paediatric HL occurs as a result of preventable or treatable conditions.^11^

Although early detection leads to appropriate interventions, hearing healthcare is inaccessible to the majority of South Africans, mainly because of lack of diagnostic equipment^12^ and scarcity of audiologists.^13^ For instance, whilst SA population exceeds 58 million, there are only 2171 audiologists registered with the Health Profession’s Council of South Africa.^13^ This compounds to a 3.7:100 000 ratio of audiologist to the population served compared to 7.3:100 000 in the United States (US).^14^ To further compound this issue, only 23% (*n* = 499) of these audiologists are currently practising in state hospitals.^13^

This scarcity of audiologists leaves people in the rural areas of SA unable to access these services. A recent report indicates that only 22 hospitals in the Eastern Cape (EC) province have 41 audiologists to render audiological services to an estimated 6.9 million people residing in the EC province. The latter compounds an estimated 0.5:100 000 ratio of audiologist to population, notwithstanding the fact that more than 30 of these audiologists are practising in urban areas. In addition to the scarcity of professionals, the majority of these hospitals do not have the necessary equipment to facilitate EDI for HL.^12^ Moreover, the recent global coronavirus disease 2019 (COVID-19) pandemic resulted in lockdown in different states globally that further restricted the access to hearing healthcare services. In SA, for instance, many public hospitals closed down non-emergency outpatient’s departments to prevent the further spread of COVID-19, which made it difficult for those with HL to access healthcare.

One of the models that may provide alternative solutions for early detection is mHealth, which is described as the use of mobile technologies to address healthcare challenges such as access, affordability and matching of resources.^15^ mHealth tools are cost effective, fast and efficient and can also be used by minimally trained people, which makes them ideal for use in cases where there is a lack of specialists.^15,16^ By using these tools, it may be possible for patients to self-screen and then be referred to the closest specialist or these results can be sent via store and send protocols, for further management. One application that may be used for EDI is the hearScreen®, which has been developed by the HearX Group, to screen for the presence of HL. Unfortunately, data on its reliability and clinical validity when patients self-administer the screening tests is unavailable. The latter is echoed by Mahomed-Asmail et al.,^17^ who argued that whilst the application may provide an alternative solution to expensive equipment, further research in its validity in children is required. As a result, alternative methods to provide hearing healthcare were needed. Therefore, this study aimed to assess the clinical validity and reliability of using self-administered smartphone applications for EDI in CLWHA.

## Methods

This feasibility study was part of a larger study that was carried out at the paediatrics department at a referral hospital in SA. After enrolling in this study, information related to their demographic features, hearing sensitivity, HIV status and HIV treatment was retrieved from the previous study’s records. To be included in this study, participants had to be aged between 6–12 years old and able to follow instructions to perform a hearing test. They were included in this study if their primary caregivers gave consent. Participants who were cognitively unable to follow instructions or those with known conditions that may affect concentration were excluded.

Non-probability purposive sampling was used to select the participants whose audiometric information was known from the larger study. Purposive sampling was used as it allowed the researchers to select participants with HL and those without HL, which enabled the researchers to assess the sensitivity and specificity of the self-administered test. Whilst the audiometric information of the participants enrolled in the study was known, the researchers were blinded to the audiological information during data collection of this study.

Since the participants’ hearing sensitivity was already known, they were given instructions to self-administer a hearing screening test using the smartphone-based hearScreen® application. The hearing screening was developed to screen for HL at four frequencies: 500 Hz- 4000 Hz at 25 dB HL. To pass the screening, participants had to hear the tone and press the button at all four test frequencies in each ear. The screening test was carried out in an ambient room using the hearScreen on a Samsung Galaxy J5 Prime smartphone and Sennheiser HD280 Pro headphones. The hearScreen® is a clinically validated tool for hearing screening with a sensitivity and specificity of > 90% when testing is administered by a hearing professional.^17^

Prior to undergoing screening, participants were given instructions in their home language and included informing them that they “should press the green button on the screen each time they heard a beeping sound in the ear”. A conditioning was done with each participant at 1000 hertz (Hz) at 40 decibels (dB) HL prior undergoing the self-administered screening. The participants underwent screening twice, with a 30-min break between the two tests.

Findings from the two self-administered tests were compared to each other to determine the test-retest reliability, sensitivity and specificity of the self-administered screening test. Moreover, self-administered results were also compared with previous audiometric information to determine correlation between the screening results with audiometric information. Statistical analysis was performed using Statistical Package for Social Sciences version 26 for Macintosh, and percentages were generated for categorical values whilst Pearson correlation coefficient assessed the test-retest reliability.

### Ethical considerations

This research adhered to the World Medical Association’s declaration of Helsinki for conducting research with human participants. Ethical approval was obtained from the University of KwaZulu-Natal’s Biomedical Research Ethics Committee (BREC/00001185/2020). All participants in this study provided assent and their caregivers gave informed consent.

## Results

A total of twenty-seven participants with a mean age of 8.3 years (range: 6–12 years) took part in this study. Eleven participants had HL whilst 16 participants had normal hearing. Table 1 summarises the demographic characteristics, HIV infection and hearing sensitivity information.

**TABLE 1:** Participant demographic characteristics.

Characteristics	Number	%	Mean
**Gender**			
Male	12	44	-
Female	15	56	-
**Age**			
6–8 years	13	47	-
9–12 years	14	53	-
**HIV infection information**			
Duration of HIV infection (years)	-	-	8.51
Duration on antiretroviral treatment (months)	-	-	16.7
CD4 count (cells/ml)	-	-	354.92
**Hearing sensitivity**			
NH	16	59	-
HL	11	41	-

NH, normal hearing; HL, hearing loss; HIV, human immunodeficiency virus.

A calculation was done to assess the sensitivity and specificity of the self-administered screening test in the detection of HL. Analysis of the results revealed a sensitivity of 82% and a specificity of 94% with positive and negative predictive values of 90% and 88%, respectively, when the test was self-administered by the participants.

Further analysis using the Pearson correlation coefficient test to assess the test-retest reliability showed a true positive test-retest reliability (*r* = 1) from the normal-hearing participants whilst a strong positive reliability (*r* = 0.98) was obtained from the participants with HL.

## Discussion

As far as we know, this was the first study to assess the sensitivity, specificity and test-retest reliability of self-administered smartphone-based hearing screening of CLWHA in SA. This study found a sensitivity and specificity of 82% and 94%, respectively, when the participants self-administered the smartphone-based hearing screening test. Moreover, these findings show that our participants reliably (*r* = 0.97) self-administered the screening. Whilst this study is the first to document sensitivity and specificity of self-administered screening, our findings are comparable to the available literature on the studies conducted amongst adult populations and those conducted by the hearing professionals. Louw et al.^16^ conducted a study at two primary health care clinics in Gauteng amongst 1236 participants aged 3–97-years-old and reported a sensitivity and specificity of 81.7% and 83.1%, respectively, when using the hearScreen® for HL detection. Another local study also found a sensitivity and specificity of 75.0% and 98.7%, respectively, when using the hearScreen® application to screen for HL in school children who are in the age group of 6–12 years.^15^ However, unlike in the present study where the participants self-administered the screening, testing in the previous two studies^15,16^ was administered by audiologists. Nevertheless, these studies consistently reported a high sensitivity and specificity of the hearScreen® to identify HL.

These findings are useful in successful planning of screening services for at-risk groups such as CLWHA. Because the hearScreen® employs artificial intelligence (AI) to allow automated testing, noise monitoring capabilities, instant data capturing and cloud-based data storage, this technology has the potential to maximise human resources where audiologists spend more time on diagnostic evaluations and treatment rather than screening. In this instance, patients can self-administer the screening and only those who fail the screening are referred to audiologists for further diagnostic evaluation and management. Moreover, these findings pave the way for decentralising hearing screening where CLWHA can self-administer the screening when they access their monthly or 3-monthly follow-up care consultations, and only those who fail the screening are referred for further diagnostic evaluation and management.

A strong positive test-retest reliability (*r* = 0.97) was obtained when the participants self-administered the hearing screening tests using the hearScreen® application. As far as we know, this was the first study to determine the test-retest reliability of self-administered screening in children, and these results should be interpreted with caution given the small sample size of this study. Nevertheless, this high test-retest reliability shows that in rural contexts such as the EC province where a scarcity of audiologists exists,^13^ the use of mobile technology such as the hearScreen®, which has AI capabilities, can improve access to EDI. However, further large-scale research, particularly on assessing the reliability of self-administration features, is highly recommended. Further application of this mHealth-supported screening model may also assist in other areas such as ototoxicity monitoring of at-risk patients undergoing treatment with known ototoxic drugs such as anti-tuberculosis drugs and platinum-based chemotherapy.

Although conventional face-to-face audiological consultations are still the status quo, alternative models of care should be employed in instances where face-to-face consultations become impossible. This is especially important in recent times where because of the global COVID-19 pandemic, travel restrictions were imposed and many public hospitals in SA closed down non-emergency outpatient’s departments, in an effort to prevent the further spread of the virus. This made it particularly difficult for those at-risk to access hearing screening. In such cases, self-administered hearing screening using the tools with AI capabilities could be used to enable the access to hearing screening for these risk groups. Self-administered screenings do not require face-to-face interactions, and thus they allow for social distancing to be maintained during assessments. Moreover, less personal protective equipment (PPE) are required because the patient does not have to be in the same room as the clinician during evaluation. Consequently, a screening/diagnostic equipment could be sent to the patient to self-administer the assessments and have the results uploaded to cloud-based storage for further analysis and treatment recommendation.

## Study limitations

The data collection was conducted when the country was under strict lockdown levels 3 and 4, during the beginning of the second wave of the COVID-19 pandemic. This imposed travel restrictions for non-emergency travel, and in addition to that, most hospitals in the country closed down non-emergency OPDs in an effort to reduce the further spread of COVID-19. These circumstances collectively reduced the number of participants who could be enrolled in this study, resulting in a small sample size in our study. Also, data collection for this study occurred in only one site, and as a result, caution should be applied when generalising these findings to the whole of the EC.

## Conclusion and recommendations

The findings from this study show a high test-retest reliability when CLWHA, who are in the age group of 6–12 years, self-administered a hearing screening test. Large-scale research is recommended to determine the feasibility of using smartphone applications to decentralise hearing screening to primary healthcare clinics where majority of the CLWHA receive their follow-up care.
